# The Relationships of Season, Climate, Habitat, and Sex Variables With Fecal Cortisol, Progesterone, and Testosterone Metabolite Concentrations in Roe Deer

**DOI:** 10.1002/ece3.73712

**Published:** 2026-05-31

**Authors:** Seyed Mehdi Amininasab, Zarbakht Ansari Pirsaraei, Ahmad Yousefpour Bisheh

**Affiliations:** ^1^ Department of Environmental Sciences and Engineering, Faculty of Natural Resources Sari Agricultural Sciences and Natural Resources University Sari Iran; ^2^ Department of Animal Sciences, Faculty of Animal Sciences and Fisheries Sari Agricultural Sciences and Natural Resources University Sari Iran; ^3^ Expert, Provincial Department of Environment of Mazandaran Province Sari Iran

**Keywords:** *Capreolus capreolus*, Cervidae, reproduction, stress

## Abstract

Monitoring cortisol, progesterone, and testosterone metabolite levels is pivotal for wildlife management, as these hormones regulate how species respond to environmental changes and reproductive functions. Environmental and biological variables can markedly alter metabolite levels, affecting the health, survival, and reproductive success of wildlife populations. This study explored how season, climate, topography, habitat quality, and sex relate to fecal cortisol, progesterone, and testosterone metabolite concentrations in roe deer ( *Capreolus capreolus* ) within Kiasar National Park and Wildlife Refuge, Mazandaran Province, northern Iran. Between 2023 and 2024, we seasonally collected 50 fresh feces samples and quantified fecal metabolite concentrations using commercial enzyme immunoassays (EIAs). We assessed habitat quality using the Normalized Difference Vegetation Index (NDVI), topography via elevation, and climate through monthly temperature and precipitation data. We then analyzed these variables, alongside sex and season, to determine their relationships with fecal metabolite concentrations. Fecal cortisol metabolite concentrations varied significantly by season, reaching their lowest levels during the summer. Males exhibited higher fecal cortisol metabolite concentrations than females; these concentrations increased with NDVI but showed no relationship with elevation, temperature, or precipitation. Fecal progesterone and testosterone metabolite concentrations varied seasonally: testosterone concentrations declined significantly in summer and autumn, while progesterone concentrations peaked in summer. Elevated NDVI and altitude reduced fecal testosterone metabolite concentrations in males, but these variables showed no relationship with fecal progesterone metabolite concentrations in females. We found no correlations between steroid hormone metabolite concentrations. These insights are crucial for informing conservation strategies, population management, and animal welfare protocols, while also advancing our understanding of roe deer evolutionary adaptations under varying environmental pressures.

## Graphical abstract

Roe deer in Kiasar National Park and Wildlife Refuge, Iran; Photo by Hamed Tizrooyan.

This study examined how seasonal, climatic, topographic, habitat, and sex variables influence fecal cortisol, testosterone, and progesterone metabolite concentrations in roe deer populations within Iran's Kiasar National Park and Wildlife Refuge through fecal sample analysis collected from 2023 to 2024. Results revealed that cortisol levels were lowest in summer and increased with habitat quality (NDVI), testosterone declined significantly during summer and autumn while being negatively affected by elevation and NDVI in males, and progesterone peaked in summer. These findings provide critical insights for developing evidence‐based conservation strategies, population management programs, and understanding adaptive responses of roe deer to environmental pressures.

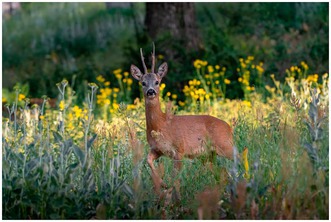

## 1 Introduction

Numerous and increasing human interventions—particularly habitat destruction, land‐use changes, and illegal hunting—heighten stress on wildlife populations, which in turn compromises their reproductive success and survival. Stress triggers the hypothalamic‐pituitary‐adrenal (HPA) axis, releasing glucocorticoids like cortisol into the bloodstream (Sapolsky et al. 2000). Physiological stress responses in wildlife vary according to intrinsic variables, such as individual physical and behavioral characteristics (Réale et al. 2007) and sex (Dziki‐Michalska et al. 2023), as well as extrinsic variables like geographic location (Robinson et al. 1965), seasonal changes, habitat quality (Fokidis et al. 2012), food availability, and regional security (Huber et al. 2003). Determining how such variables affect glucocorticoids remains difficult (Bhaskaran et al. 2026), as measuring glucocorticoids does not equate to measuring a stress response (MacDougall‐Shackleton et al. 2019) and requires rigorous, species‐ and matrix‐specific validation (Palme 2019). Furthermore, numerous uncontrolled variables shape HPA activity (Bhaskaran et al. 2026). Evolution co‐opted the signaling functions of these metabolic hormones into a diverse and integrated stress response (MacDougall‐Shackleton et al. 2019). Monitoring these indicators provides crucial insights into wildlife physiology, growth, body condition, fertility, social behavior, and survival (Boonstra 2005). Quantifying stress indicators offers insights into wildlife health while improving our ecological and evolutionary understanding for conservation and management. Researchers widely use cortisol or corticosterone metabolite concentrations as stress indicators to reveal how wildlife respond to natural stressors (e.g., climate, breeding season territoriality) and human‐induced ones, such as habitat loss and land‐use changes.

Reproduction remains a critical phase in wildlife life cycles, driving population renewal and survival (Pavitt et al. 2016). Before the breeding season, female ovarian activity begins with rising levels of sex steroid hormones, including progesterone, while male testicular activity intensifies through increased testosterone production (Asher 2010). Testosterone levels prove physiologically crucial for male reproduction, particularly during mating season competition (Peters et al. 2008). Understanding these metabolite levels and their relationship to habitat, climate, season, physiology, stress, and human interventions (Cumming et al. 1983) significantly informs conservation and management decisions.

Among sampling techniques, feces sampling offers a non‐invasive, stress‐free, and safe method for measuring hormones in herbivores (Dziki‐Michalska et al. 2023; Grotta‐Neto et al. 2024). Fecal metabolite levels serve as reliable markers for assessing stress and reproduction in wildlife (Palme et al. 2005; Dantzer et al. 2014).

Large herbivores face particular vulnerability to environmental stress and unpredictable changes due to their extensive home ranges (Romero and Wingfield 2016). Deer, with their territorial behaviors, serve as suitable indicators for monitoring environmental changes and habitat health (Hanley 1996; Vanp'e et al. 2009). Among these species, the roe deer ( *Capreolus capreolus* )—the smallest cervid and a medium‐sized mammal (Lee and Rezaie 2021)—inhabits a wide range across Palearctic climates (Evcin et al. 2019). The IUCN Red List categorizes it as “Least Concern” due to its broad distribution (IUCN 2018). Habitat destruction and excessive hunting pose the primary threats to this species (Karami et al. 2015). Unfortunately, increasing human interventions and stressors—such as road construction and forest exploitation—now threaten the Hyrcanian forests. Given the roe deer's high vulnerability to human‐altered habitats (Padie et al. 2015), researchers must prioritize measuring stress and sex hormone metabolite concentrations in these populations. Investigating how season, climate, topography, habitat quality, and sex relate to fecal cortisol, progesterone, and testosterone metabolite concentrations identifies existing threats, assesses reproductive status, and helps prioritize conservation efforts for roe deer.

## 2 Materials and Methods

### 2.1 Study Area

Kiasar National Park, a mountainous region in the central Alborz Mountains of Mazandaran Province, northern Iran, lies approximately 70 km south of Sari. Spanning 9530 ha, the park sits between 53°28′20′′ and 53°41′21′′ E and 36°7′38′′ and 36°15′10′′ N, about 10 km south of Kiasar (Kanaani 2006). The area receives an average annual precipitation of 650 mm and maintains a mean annual temperature of 12°C. Elevations within the park range from 883 m to 2775 m above sea level.

The Dodangeh‐Chahardangeh Wildlife Refuge covers 16,869 ha and sits 95 km southeast of Sari in northern Iran, between 53°23′ and 53°40′ E and 36°4′ and 36°10′ N (Moasagh 2015). Elevations in the region range from 800 m to 2860 m. A cold, humid climate prevails between 800 and 2000 m, while semi‐arid, cold conditions characterize areas above 2000 m. Average annual precipitation reaches 643 mm at lower elevations and 253 mm at higher altitudes (Moasagh 2015).

Kiasar National Park and the Wildlife Refuge feature a temperate semi‐humid climate. These forested, rocky‐mountainous regions in the mid‐ and upper belts of the Hyrcanian forests provide habitats for unique forest species. The western and northern regions function as a biosphere reserve, while the eastern and southern regions serve as a national park (Kanaani 2006; Moasagh 2015).

Key mammal species in the region include *Capreolus capreolus* , *Cervus elaphus* , *Panthera pardus saxicolor* , *Ursus arctos* , *Sus scrofa* , *Canis aureus* , *Vulpes vulpes* , *Felis chaus* , *Felis silvestris* , *Mustela nivalis* , *Martes foina* , and *Hystrix indica* . High biodiversity, pristine Hyrcanian forests, and these valuable indicator mammals make this park environmentally unique (Kanaani 2006; Moasagh 2015).

### 2.2 Feces Sampling and Storage

Two observers searched for roe deer feces samples using random walks across 5‐m‐wide transects. They focused on locations where environmental guards had previously observed deer during daily monitoring in Kiasar National Park and the Wildlife Refuge. Fieldwork spanned four consecutive days each month for 1 year (2023–2024), covering all four seasons, with observers searching for 12 h daily starting at 6:00 AM.

After visually scanning and locating feces presence points, we collected 50 fresh samples. Based on moisture and a glossy appearance, we selected only samples excreted within the previous 4 h. If we encountered multiple pellet groups at a single location, we gathered only one. We then placed the samples in labeled plastic bags, kept them on ice for up to 4 h, and stored them in a freezer at −20°C until analysis (Netto et al. 2000). Because this study relied solely on non‐invasive, stress‐free feces sampling—a method that prevents mortality in herbivore populations (Cornelis et al. 1999)—it did not require ethical approval.

### 2.3 Sex Determination

We determined the sex of the roe deer concurrently with sample collection based on the shape and appearance of the pellets. Male pellet groups typically feature larger, sharper edges, while female pellets are rounder. Although genetic testing confirms sex with certainty (Aznar‐Cormano et al. 2021), resource limitations required us to estimate sex by measuring fecal testosterone and progesterone metabolite concentrations. This method relies on the higher fecal metabolite concentrations in their respective sexes (testosterone in males and progesterone in females).

### 2.4 Collection of Climatic and Ecological Data

We identified roe deer feces pellet group locations and recorded their geographic coordinates using GPS. Following sampling, we obtained climatic variables—including temperature, precipitation, humidity, and sunshine hours—from the nearest meteorological station (Wasser et al. 2000; Huber et al. 2003). Among the ecological variables, we selected elevation to represent topography and the Normalized Difference Vegetation Index (NDVI) as a habitat indicator. We generated elevation data using the Shuttle Radar Topography Mission (SRTM DEM) digital elevation model with 30‐m spatial resolution and used Landsat 8 satellite imagery to produce the NDVI data (Roy and Zhang 2019).

### 2.5 Measurement of Fecal Cortisol and Sex Hormone Metabolite Concentrations

For measuring fecal cortisol and sex hormone metabolite concentrations, we moved frozen feces samples from the freezer and dried them in an oven at 50°C–60°C for 48 h. We then ground the dried samples using a mortar and pestle and filtered them through a 1‐mm sieve. Next, we took 0.5 g of the feces, added 2 mL of phosphate buffer and 2 mL of 100% methanol, and mixed it for 16 h on a shaker. We centrifuged the test tube for 30 min at 4000 rpm.

We prepared the samples using AccuBind enzyme immunoassay (EIA) kits (Monobind Inc.; codes 3625‐300 for cortisol, 3725‐300 for testosterone, and 4825‐300 for progesterone; Table S4) according to the manufacturer's instructions (see Yousefpour Bisheh et al. 2025). The kits included: a microplate with 96 wells of immobilized streptavidin; calibrator vials with specific concentrations (testosterone: 0–12 ng/mL; progesterone: 0–60 ng/mL; cortisol: 0–50 μg/dL); and solutions for enzyme conjugate, washing, substrate A and B, and stopping the reaction. We added 25 μL of standards, control samples, and selected samples for progesterone and cortisol, and 10 μL for testosterone, to the designated wells. Using separate kits for each hormone, we added 100 μL of the enzyme conjugate solution to the wells. We gently shook the plate for 20–30 s to mix the conjugate with the samples and covered the wells with adhesive film to prevent evaporation during incubation. After incubating the plate for 60 min at room temperature (22°C), we emptied the wells and washed them three times with at least 350 μL of wash buffer per well. To ensure complete removal of the buffer, we firmly tapped the plate on absorbent paper. We then added 100 μL of prepared substrate solution (50 μL each of Chromogen A and B) to each well and incubated the plate in the dark for 15 min to protect the light‐sensitive chromogen. Finally, we added 50 μL of stop solution to each well, shook the plate for 15–20 s, and read the absorbance at 450 nm (using a 630 nm reference wavelength). We ensured the reading occurred within 30 min of adding the stop solution. The ELISA reader generated standard curves to calculate hormone concentrations; we expressed fecal testosterone and progesterone metabolite concentrations in ng and fecal cortisol metabolite concentrations in μg per g feces (Abdul Hamid et al. 2022).

### 2.6 Statistical Analysis

We analyzed the data using R software and report the results as mean values and standard deviations. We set the significance level at 0.05. After confirming that the data followed a normal distribution, we used multivariate regression to build a maximal model. This model included independent and predictor variables—season, climate, habitat quality, and sex—with roe deer fecal metabolite concentrations (cortisol, progesterone, and testosterone) serving as separate dependent variables.

We examined correlations between these variables using principal component analysis (PCA) and Pearson correlation tests, including only those without high correlations in the maximal model. Using stepwise backward selection with the “step” function, we removed variables with the highest non‐significant values to produce a final minimal model containing only statistically significant relationships. Finally, we used Pearson correlation tests to identify significant correlations between the different fecal metabolite concentrations.

## 3 Results

### 3.1 Descriptive Statistics of Climatic, Topographic, and Habitat Variables Across Study Seasons

We provided monthly averages for air temperature, precipitation, humidity, and sunshine hours (Table S1). After evaluating correlations using principal component analysis and Pearson coefficients, we selected average monthly air temperature and precipitation as representative climatic variables (Figure S1, Table S2). We also presented seasonal statistics for elevation and NDVI (Table S3), and fecal testosterone, progesterone, and cortisol metabolite concentrations (Table 1).

**TABLE 1:** Mean and standard deviation of fecal testosterone and progesterone (ng per g feces) and cortisol (μg per g feces) metabolite concentrations in roe deer, categorized by sex and season.

Season	Sex	Cortisol		Testosterone		Progesterone		Sample size
		Mean	Standard deviation	Mean	Standard deviation	Mean	Standard deviation	
Spring	Male	0.47	0.05	109.87	10.19	5.87	1.67	3
	Female	0.57	0.01	4.00	0.80	125.60	0.80	3
Summer	Male	0.46	0.01	72.80	0.80	7.20	0.80	3
	Female	0.50	0.07	4.00	1.46	126.40	24.47	4
Autumn	Male	0.62	0.05	79.92	10.06	6.40	1.92	10
	Female	0.53	0.03	5.33	1.20	105.47	11.20	6
Winter	Male	0.65	0.08	76.74	8.58	7.45	2.13	13
	Female	0.56	0.04	5.70	1.51	97.00	12.07	8

### 3.2 The Relationships Between Climatic, Topographic, Habitat, Sex, and Seasonal Variables With Roe Deer Fecal Metabolite Concentrations

**FIGURE 1:**
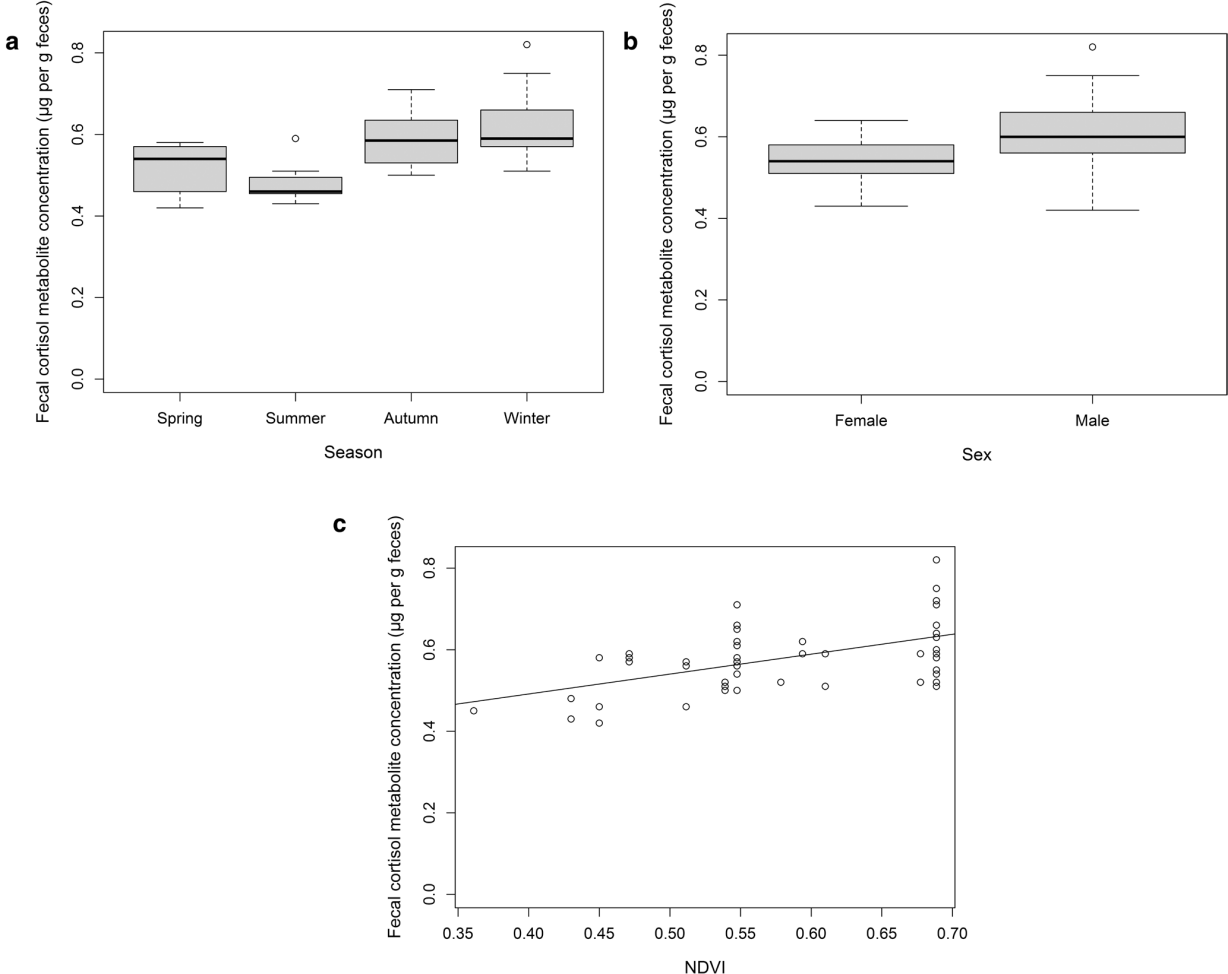
(a) Roe deer fecal cortisol metabolite concentrations varied significantly by season, reaching a minimum during the summer. (b) Male roe deer exhibited higher fecal cortisol metabolite concentrations than females. (c) Fecal cortisol metabolite concentrations rose significantly as the NDVI index increased. Panels (a) and (b) show the minimum, maximum, and median confidence intervals, which represent the median range ±1.57 times the interquartile range (first to third quartile).

We presented the relationships between roe deer fecal cortisol metabolite concentrations and climatic variables (temperature and precipitation), topography (elevation), habitat (NDVI index), sex, and season (Table 2). The final model identified season as a significant variable for fecal cortisol metabolite concentrations, which reached their minimum in summer (Table 2, Figure 1a). Sex also related to these concentrations; males maintained higher fecal cortisol metabolite concentrations than females (Table 2, Figure 1b). Moreover, fecal cortisol metabolite concentrations increased significantly alongside rising NDVI values (Table 2, Figure 1c).

**TABLE 2:** Relationship of climatic variables (temperature and precipitation), topography (elevation), habitat (NDVI), sex, and season to fecal cortisol metabolite concentration (μg per g feces) in roe deer.

	Estimate	Standard error	*t*	*p*
**Maximal model**				
Intercept	0.394	0.399	0.990	0.328
Temperature	0.008	0.009	0.910	0.368
Precipitation	0.000	0.000	0.468	0.643
Elevation	−0.000	0.000	−0.330	0.743
NDVI	0.160	0.463	0.347	0.731
Spring season	−0.071	0.061	−1.174	0.247
Summer season	−0.126	0.127	−0.998	0.324
Winter season	0.062	0.050	1.226	0.227
Male gender	0.041	0.022	1.844	0.072
**Minimal model**				
Intercept	0.388	0.079	4.878	≤ 0.001***
Male gender	0.050	0.019	2.694	≤ 0.01**
Summer season	−0.072	0.032	−2.274	0.027*
NDVI	0.305	0.139	2.193	0.034*

*Note:* *p ≤ 0.05, **p ≤ 0.01, ***p ≤ 0.001.

**FIGURE 2:**
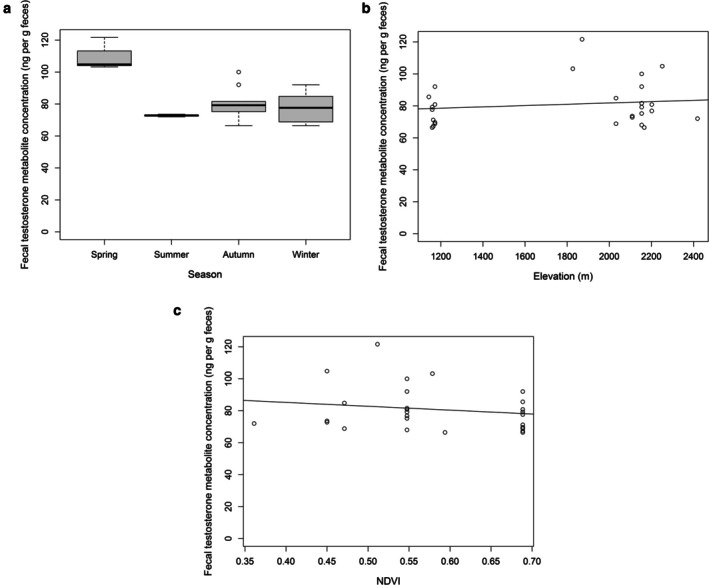
(a) Male roe deer exhibited significantly lower fecal testosterone metabolite concentrations during summer and winter. Panel (a) presents the minimum, maximum, and median confidence intervals based on the median range ±1.57 times the interquartile range (first to third quartile). (b) Fecal testosterone metabolite concentrations in male roe deer rose as elevation increased. (c) Fecal testosterone metabolite concentrations in male roe deer fell as the NDVI index increased.

We explored the relationship between climatic variables (temperature and precipitation), topography (elevation), habitat (NDVI index), season, and fecal testosterone metabolite concentrations in male roe deer (Table 3). The final model identified elevation, NDVI, and season as significant variables, with concentrations dropping during summer and winter (Table 3, Figure 2a). Specifically, fecal testosterone metabolite concentrations declined with increasing elevation and NDVI values (Figures 2b, 2c).

**TABLE 3:** Relationship of climatic variables (temperature and precipitation), topography (elevation), habitat (NDVI), and season to fecal testosterone metabolite concentration (ng per g feces) in male roe deer.

	Estimate	Standard error	*t*	*p*
**Maximal model**				
Intercept	527.471	223.010	2.365	0.028
Temperature	−2.222	3.336	−0.666	0.513
Precipitation	0.125	0.195	0.641	0.529
Elevation	−0.106	0.049	−2.189	0.040
NDVI	−358.904	165.610	−2.167	0.042
Spring season	3.801	16.138	0.236	0.816
Summer season	−26.901	33.289	−0.808	0.428
Winter season	−63.263	43.116	−1.467	0.157
**Minimal model**				
Intercept	446.328	144.592	3.087	0.005
Elevation	−0.085	0.034	−2.519	0.019*
NDVI	−331.616	131.406	−2.524	0.019*
Summer season	−46.792	16.664	−2.808	0.009**
Winter season	−42.592	16.558	−2.572	0.017*

*Note:* *p ≤ 0.05, **p ≤ 0.01, ***p ≤ 0.001.

**FIGURE 3:**
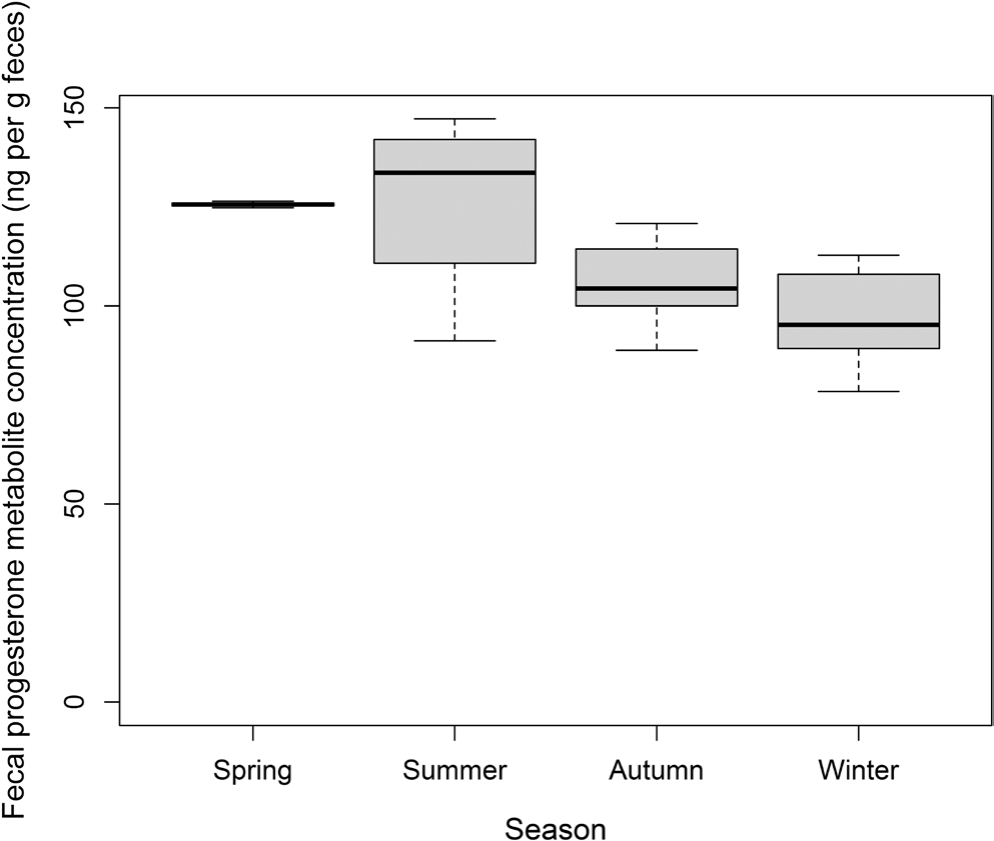
Fecal progesterone metabolite concentrations in female roe deer varied significantly across seasons, with levels peaking during the summer. The figure shows the minimum, maximum, and median confidence intervals based on the median range ±1.57 times the interquartile range (first to third quartile).

Finally, we presented the relationships between climatic variables (temperature and precipitation), topography (elevation), habitat (NDVI index), and female fecal progesterone metabolite concentrations (Table 4). Our analysis revealed a significant relationship between season and fecal progesterone metabolite concentrations, which peaked during the summer (Table 4, Figure 3).

**TABLE 4:** Relationship of climatic variables (temperature and precipitation), topography (elevation), habitat (NDVI), and season to fecal progesterone metabolite concentration (ng per g feces) in female roe deer.

	Estimate	Standard error	*t*	*p*
**Maximal model**				
Intercept	4486	3371	1.331	0.206
Temperature	−898	660.8	−1.359	0.197
Precipitation	77.85	57.79	1.347	0.201
Elevation	0.113	0.056	2.019	0.065
NDVI	507.3	232.5	2.182	0.048
Spring season	3979	2902	1.371	0.194
Summer season	11,560	8494	1.361	0.197
Winter season	1690	1272	1.328	0.207
**Minimal model**				
Intercept	105.467	5.811	18.148	≤ 0.001***
Summer season	20.933	9.189	2.278	0.036 *

*Note:* *p ≤ 0.05, **p ≤ 0.01, ***p ≤ 0.001.

### 3.3 The Correlations Between Fecal Cortisol, Testosterone, and Progesterone Metabolite Concentrations in Roe Deer

Pearson correlations revealed no significant relationship between fecal cortisol and testosterone metabolite concentrations in males (*p* = 0.373, df = 27, *r* = −0.172) or between fecal cortisol and progesterone metabolite concentrations in females (*p* = 0.761, df = 19, *r* = −0.070).

## 4 Discussion

Roe deer fecal cortisol metabolite concentrations varied significantly by season, peaking in winter and reaching a minimum in summer. However, these differences lacked statistical significance, except for the summer, when concentrations dropped significantly below those of other seasons. This suggests that roe deer experience less stress during summer, likely due to seasonal variations in natural and human‐induced stressors. The elevated stress during winter and autumn likely stems from lower air temperatures, regional snow cover, limited food resources, and cold‐induced reductions in metabolic rates (Küker et al. 2015).

Although one might expect higher fecal cortisol metabolite concentrations in summer due to breeding‐season stressors, our findings revealed lower concentrations during this period compared to other seasons. This suggests that greater food availability and higher temperatures in summer and spring likely reduce stress levels. Our findings align with Ventrella et al. (2018), who observed no significant differences in blood cortisol concentrations before or after the breeding season in Italian roe deer. Conversely, Dziki‐Michalska et al. (2023) reported significant summer increases in cortisol metabolite concentrations among Polish roe deer, attributing the higher stress to the mating season. In Spain, Escribano‐Avila et al. (2013) found that cortisol metabolite concentrations peaked in spring and reached their minimum in autumn. Nonetheless, various variables unrelated to reproductive physiology, including diverse natural and human‐induced seasonal stressors, influence wildlife stress (Young et al. 2017). Some studies show that roe deer maintain stable stress indicators even when facing human disturbances or a lack of predators (Zbyryt et al. 2017). Conversely, other research indicates that environments near human infrastructure likely increase stress in roe deer and other cervids (Bonnot et al. 2018; Carbillet et al. 2020).

Temperature and precipitation showed no relationship with fecal cortisol metabolite concentrations, suggesting that climate does not strongly drive stress in this roe deer population. This indicates either minimal fluctuations in temperature and monthly precipitation within the sampling areas or a high degree of physiological adaptation among roe deer to their local climate. Contrary to our results, French roe deer exhibited higher fecal cortisol metabolite concentrations at lower temperatures (Carbillet et al. 2020). While our data showed higher fecal cortisol metabolite concentrations in winter and autumn compared to summer and spring, these differences lacked statistical significance.

Males maintained significantly higher fecal cortisol metabolite concentrations than females. Similarly, Dziki‐Michalska et al. (2023) found sex‐dependent cortisol concentrations in Poland, noting higher concentrations in males due to territorial pressures. This suggests that reproductive and physiological status influences stress levels and subsequent cortisol metabolite concentrations (He et al. 2014). In contrast, Küker et al. (2015) found no significant sexual dimorphism in cortisol concentrations among Swedish roe deer, suggesting both sexes faced similar environmental stressors. Notably, female deer often exhibit higher cortisol concentrations due to the stress of pregnancy and lactation (He et al. 2014; Pavitt et al. 2016); however, we did not observe this trend in our study population.

Our results show that habitat quality (measured by the NDVI) significantly relates to fecal cortisol metabolite concentrations, with roe deer likely experiencing higher stress in more vegetated areas. Research indicates that roe deer in open areas with sparse vegetation face higher stress, particularly near human infrastructure or during hunting seasons (Martin et al. 2018; Carbillet et al. 2020). Despite the expected role of vegetation in providing food and shelter (Carbillet et al. 2020), our findings revealed an inverse relationship. This outcome may result from stable NDVI values across sampling areas or high physiological adaptation to vegetation changes, allowing roe deer to meet their needs through grazing and movement (Ferretti et al. 2015; Carbillet et al. 2020). Nonetheless, the higher stress observed in densely vegetated areas—mirroring some other studies (Carbillet et al. 2020)—may stem from unmeasured environmental variables or unknown mechanisms that our study could not isolate.

Elevation did not significantly relate with fecal cortisol metabolite concentrations, suggesting that roe deer stress levels remain independent of this topographic variable. This likely reflects minimal elevation changes across sampling sites or a high capacity for roe deer to adapt to altitudinal shifts without experiencing stress.

Environmental and habitat conditions primarily drive cortisol metabolite concentrations in deer species (Ventrella et al. 2020). Research on French roe deer showed higher cortisol concentrations during years with poor resource quality (Carbillet et al. 2020). Consequently, environmental changes and pressures from natural or anthropogenic variables (Caslini et al. 2016) may stimulate cortisol secretion, causing varied behavioral and physiological responses within and across deer species (He et al. 2014; Dziki‐Michalska et al. 2023). Comparative analyses must also account for the specific tissue analyzed (e.g., feces, hair, or blood plasma), as correlations between cortisol concentrations in different tissues often vary, even within the same species (Vilela et al. 2020).

Fecal testosterone metabolite concentrations varied significantly by season, dropping during summer and winter. Conversely, fecal progesterone metabolite concentrations peaked significantly during the summer months. Seasonal shifts in temperature, precipitation, and day length influence the secretion of reproductive hormones and the timing of the breeding season (Gordon 1997). Temperature and precipitation showed no relationship with fecal sex hormone metabolite concentrations, suggesting that seasonal fluctuations follow changes in environmental resources and food accessibility (Haigh and Hudson 1993). Nonetheless, consistent with previous research, fecal testosterone and progesterone metabolite concentrations differed significantly during the summer breeding season, when females exhibited higher progesterone (Blottner et al. 1996; Roelants et al. 2002; Kozioł and Koziorowski 2013). Some studies show that testosterone concentrations reach their minimum in autumn and peak in spring, just before the rutting season (Escribano‐Avila et al. 2013). Progesterone plays a vital role in fetal protection and mammary development during winter pregnancy and spring lactation (Ryg 1986). Consequently, male fecal testosterone metabolite concentrations declined in winter, spring, and summer after the mating season, aligning with our results.

Male fecal testosterone metabolite concentrations decreased significantly at higher elevations. This suggests that lower metabolic rates or other environmental mechanisms reduce testosterone in high‐altitude males, whereas more capable males may prefer lower elevations.

Habitat quality, as measured by the NDVI, also showed a significant relationship with fecal testosterone metabolite concentrations. Differences in habitat quality and food access typically influence hormone levels (De la Peña et al. 2021). While dense vegetation provides shelter and enhances mating opportunities or territorial defense (Gomez et al. 2006; McPhee and Carlstead 2010; Mateos‐Quesada 2011), potentially boosting reproductive hormones (De la Peña et al. 2021), our findings differed. Contrary to expectations, female fecal progesterone metabolite concentrations showed no relationship with vegetation density, and males in denser vegetation exhibited lower fecal testosterone metabolite concentrations. This suggests that unmeasured environmental variables or unknown mechanisms operate in highly vegetated habitats, making it difficult to isolate the specific role of vegetation on male testosterone.

Pearson correlations revealed no significant relationship between fecal cortisol metabolite concentrations and either testosterone in males or progesterone in females. This lack of correlation suggests that the HPA and gonadal axes operate independently, likely driven by distinct seasonal and environmental variables (Sirotkin et al. 2016; Ventrella et al. 2018). Long‐term reproductive cycles and photoperiodic triggers primarily dictate sex hormone levels rather than acute, cortisol‐mediated stress (Schams and Barth 1982; Sempéré et al. 1992). Cortisol concentrations typically respond to immediate metabolic demands or environmental stressors that may not overlap with stable reproductive states (Sempéré and Boissin 1981; Ventrella et al. 2018). Additionally, fecal metabolite levels reflect hormonal activity over varied timeframes, which can decouple the presentation of stress and reproductive markers in excreta (Ventrella et al. 2018). Consequently, these results imply that reproductive physiology and stress responses function independently in both sexes during the study period (Sirotkin et al. 2016). In contrast, research on Italian roe deer showed that rising cortisol concentrations led to decreased testosterone (Escribano‐Avila et al. 2013; Ventrella et al. 2018). Escribano‐Avila et al. (2013) also found that progesterone correlated positively with cortisol only during winter and spring (the rutting and lactation seasons). Several wildlife studies demonstrate inverse relationships between cortisol and sex hormones, indicating that stress metabolites suppress glandular function (Wingfield et al. 1994; Schuett et al. 1996; Blanchard et al. 2001).

## 5 Limitations and Suggestions

Sampling free‐ranging roe deer across 26,399 ha of natural habitat posed significant logistical challenges. Despite conducting multiple monthly surveys each season, we collected only 50 feces samples during the one‐year study. We intensified our sampling in spring and summer; however, dense vegetation and rapid shrub growth during these months made locating feces exceptionally difficult. This small sample size reflects the inherent difficulties of field research on wild populations rather than a lack of effort. Furthermore, we did not validate which EIA detects expected increases in roe deer fecal cortisol metabolite concentrations after a stressful event (see Palme 2019), a shortcoming of the current study. Future experimental studies (Bhaskaran et al. 2026) with larger seasonal sample sizes and biological validation methods will likely better elucidate how environmental and biological variables influence fecal sex hormone and stress metabolite concentrations.

Overall, one should expect contradictory findings across roe deer studies regarding how various variables influence fecal metabolite concentrations. Relying solely on glucocorticoids as stress indicators limits our understanding of how species respond to ecological, social, and environmental pressures (MacDougall‐Shackleton et al. 2019; Lipowska et al. 2022). Researchers must therefore analyze these relationships within the context of ecological variables like habitat and climate, geographic latitude (Santos et al. 2018; Grotta‐Neto et al. 2024), animal density (Seal et al. 1983), reproductive stages (Pavitt et al. 2016; Dziki‐Michalska et al. 2023), tissue types (Palme 2019; Vilela et al. 2020), animal age (Fehér et al. 1994; Pavitt et al. 2016; Santos et al. 2018; Carbillet et al. 2019; Dziki‐Michalska et al. 2023), animal body condition or social rank (Pavitt et al. 2016; De la Peña et al. 2021), animal species and behavior (Palme 2019; Lipowska et al. 2022; Dziki‐Michalska et al. 2023), and the context of species‐ and matrix‐specific validation (Palme 2019). Nonetheless, identifying significant drivers of hormone levels informs conservation strategies and management decisions regarding population dynamics (Carbillet et al. 2020), reproductive status (Dziki‐Michalska et al. 2023), energy costs (De la Peña et al. 2021), welfare (Carbillet et al. 2019), and evolutionary responses to human activity (Santos et al. 2018).

## 6 Conclusions

Fecal cortisol metabolite concentrations in roe deer varied significantly across seasons, with lower summer levels likely reflecting reduced stress. Climatic variables, such as temperature and precipitation, showed no relationship with these concentrations. Male roe deer exhibited higher fecal cortisol metabolite concentrations than females, and individuals in areas with dense vegetation showed higher concentrations. However, fecal cortisol metabolite concentrations showed no relationship with elevation.

Fecal testosterone and progesterone metabolite concentrations varied significantly across seasons; testosterone concentrations dropped in summer and autumn, while progesterone concentrations peaked in summer. In males, fecal testosterone metabolite concentrations decreased as elevation and vegetation density increased. Conversely, female fecal progesterone metabolite concentrations showed no relationship with elevation or vegetation. Finally, no correlation existed between cortisol and either fecal testosterone or progesterone metabolite concentrations in either sex.

## 7 Conservation and Management Applications

This study demonstrates how season, sex, vegetation, and elevation relate to fecal cortisol, testosterone, and progesterone metabolite concentrations in roe deer. Conservationists and wildlife managers can apply these findings to roe deer management in the following ways:

- Seasonal management and activity timing: The findings show that roe deer have lower fecal cortisol metabolite concentrations in summer, which likely indicates reduced stress during this season. Managers should therefore schedule habitat restoration, population monitoring, or human activity control during these low‐stress periods to avoid placing additional physiological burdens on the animals (Sheriff et al. 2011). Conversely, higher stress levels in other seasons necessitate reducing disturbances during those times to improve animal welfare and reproductive success.
- Attention to sexual differences in stress management: Higher fecal cortisol metabolite concentrations in males and distinct hormonal responses between the sexes underscore the importance of sex‐specific management. Because stress may increase fecal testosterone metabolite concentrations and male sexual performance, it could alter mating dynamics; planners must consider this when designing population control or breeding programs.
- Vegetation and habitat quality management: The link between dense vegetation and higher fecal cortisol metabolite concentrations, along with the drop in male fecal testosterone metabolite concentrations as elevation and vegetation density increase, highlights the complex role of habitat structure. While dense vegetation provides shelter, it likely increases competition or predation risk, which elevates stress (Boonstra et al. 1998). Managers should create diverse, balanced habitats that offer both shelter and sufficient resources to optimize the animals' physiological health.

By leveraging these findings, wildlife managers can develop more targeted and effective conservation programs to sustain roe deer populations and ensure healthy physiological and population dynamics.

## Author Contributions

**Seyed Mehdi Amininasab:** conceptualization (lead), data curation (equal), formal analysis (lead), funding acquisition (lead), investigation (lead), methodology (equal), project administration (lead), validation (equal), visualization (lead), writing – original draft (lead), writing – review and editing (lead). **Zarbakht Ansari Pirsaraei:** data curation (equal), investigation (equal), methodology (equal), validation (equal), visualization (equal). **Ahmad Yousefpour Bisheh:** data curation (equal), investigation (equal), methodology (equal), validation (equal), visualization (equal).

## Funding

This research project has been financially supported through No. 5822/1402, dated August 31, 2023, by the Department of environmental conservation of Mazandaran province in collaboration with Sari Agricultural Sciences and Natural Resources University.

## Conflicts of Interest

The authors declare no conflicts of interest.

## Supporting information


**TABLE S1:** Descriptive statistics for monthly averages of air temperature, precipitation, humidity percentage, and sunshine hours across the four sampling seasons.
**TABLE S2:** Pearson correlation coefficients for climatic variables (values represent correlation coefficients and asterisks denote significance levels).
**TABLE S3:** Descriptive statistics for elevation and NDVI across different seasons.
**Table S4:** Sensitivity, precision, and cross‐reactivity for cortisol, testosterone, and progesterone AccuBind enzyme immunoassay (EIA) kits (Monobind Inc.; codes 3625–300 for cortisol, 3725‐300 for testosterone, and 4825–300 for progesterone).
**FIGURE S1:** Principal component analysis (PCA) for climatic variables (air temperature, precipitation, humidity percentage, and sunshine hours).

## Data Availability

The data for this study are available on: https://datadryad.org/dataset/doi:10.5061/dryad.ngf1vhj8w.
