# Supplementary material for: The Relationships of Season, Climate, Habitat, and Sex Variables With Fecal Cortisol, Progesterone, and Testosterone Metabolite Concentrations in Roe Deer
Source: Ecol Evol. 2026 May 31;16(6):e73712. doi: 10.1002/ece3.73712 (PMC13240073; doi:10.1002/ece3.73712)
Supplement: Supplementary file 1 — TABLE S1: Descriptive statistics for monthly averages of air temperature, precipitation, humidity percentage, and sunshine hours across the four sampling seasons. TABLE S2: Pearson correlation coefficients for climatic variables (values represent correlation coefficients and asterisks denote significance levels). TABLE S3: Descriptive statistics for elevation and NDVI across different seasons. Table S4: Sensitivity, precision, and cross‐reactivity for cortisol, testosterone, and progesterone AccuBind enzyme immunoassay (EIA) kits (Monobind Inc.; codes 3625–300 for cortisol, 3725‐300 for testosterone, and 4825–300 for progesterone). FIGURE S1: Principal component analysis (PCA) for climatic variables (air temperature, precipitation, humidity percentage, and sunshine hours). [file ECE3-16-e73712-s001.pdf]

Supporting Information

TABLE S1. Descriptive statistics for monthly averages of air temperature, precipitation, humidity percentage, and sunshine hours across the four sampling seasons.

Parameter	Season	Mean
**Air temperature**	Spring	16.49
	Summer	20.77
	Autumn	14.20
	Winter	8.34
**Precipitation**	Spring	77
	Summer	29.4
	Autumn	100.84
	Winter	30.64
**Humidity**	Spring	73
**percentage**	Summer	79
	Autumn	64.19
	Winter	28.65
**Sunshine hours**	Spring	215.13
	Summer	153.30
	Autumn	185.58
	Winter	190.73

TABLE S2. Pearson correlation coefficients for climatic variables (values represent correlation coefficients and asterisks denote significance levels).

	**Precipitation**	**Humidity percentage**	**Sunshine hours**
**Air temperature**	0.42 **	0.74 ***	-0.10
**Precipitation**		-0.007	0.19
**Humidity percentage**			-0.10

TABLE S3. Descriptive statistics for elevation and NDVI across different seasons.

Parameter	Season	Mean	Standard deviation	Sample size
**Elevation**	Spring	1990.67	203.14	6
	Summer	1859.57	441.90	7
	Autumn	2085	177.08	16
	Winter	1315.667	357.29	21
**Vegetation NDVI**	Spring	0.50	0.05	6
	Summer	0.48	0.09	7
	Autumn	0.55	0.02	16
	Winter	0.64	0.09	21

Table S4. Sensitivity, precision, and cross-reactivity for cortisol, testosterone, and progesterone AccuBind enzyme immunoassay (EIA) kits (Monobind Inc.; codes 3625-300 for cortisol, 3725-300 for testosterone, and 4825-300 for progesterone).

**Kit type**	**Sensitivity**	**Precision (%)**	**Cross Reactivity with other steroids (%)**
Cortisol	0.366 µg/dl	Intra-assay CV<br>Low Sample: 8.2<br>Normal sample: 6.4<br>High sample: 6.1<br><br>Inter-assay CV<br>Low Sample: 9.7<br>Normal sample: 7.0<br>High sample: 7.3	Cortisol:100<br>Cortisone: 23<br>Corticosterone: 18<br>11-Deoxycortisol: 5.50<br>Androstenedione: 0.04<br>Progesterone: 0.02<br>Dexamethasone: 0.01<br>17α-oh progesterone: ND<br>DHEA: ND<br>Estradiol: ND<br>Estrone: ND<br>Danazol: ND<br>Testosterone: ND
Testosterone	0.0576 ng/ml	Intra-assay CV<br>Low Sample: 9.8<br>Normal sample: 4.8<br>High sample: 5.6<br><br>Inter-assay CV<br>Low Sample: 9.1<br>Normal sample: 9.7<br>High sample: 7.9	Testosterone: 100<br>Dihydrotestosterone: 1.78<br>Androstenedione: 0.09<br>Cortisone: < 0.01<br>Corticosterone: < 0.01<br>Cortisol: < 0.01<br>Progesterone: < 0.01<br>17α-oh progesterone: < 0.01<br>DHEA sulfate: < 0.01<br>Estradiol: < 0.01<br>Estrone: < 0.01<br>Estriol: < 0.01
Progesterone	0.105 ng/ml	Intra-assay CV<br>Low Sample: 15.3<br>Normal sample: 3.8<br>High sample: 6.1<br><br>Inter-assay CV<br>Low Sample: 8.9<br>Normal sample: 7.5<br>High sample: 6.4	Progesterone: 100<br>17oh-progesterone: 0.375<br>Corticosterone: 0.347<br>Androstenedione: 0.158<br>Prednisone: 0.023<br>Testosterone: 0.015<br>Cortisone: 0.014<br>Dihydrotestosterone: 0.006<br>Cortisol: 0.005<br>Estradiol: 0.004<br>Danazol: 0.003<br>Estrone: 0.003<br>DHEA sulfate: 0.002<br>Estriol: 0.002

ND: None Detected

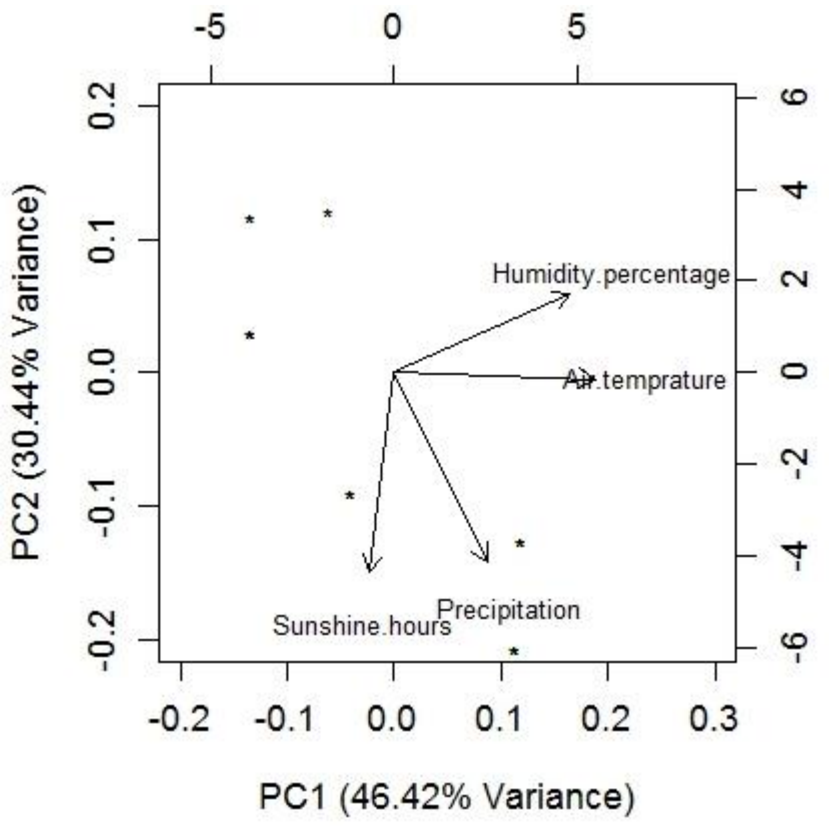

FIGURE S1. Principal component analysis (PCA) for climatic variables (air temperature, precipitation, humidity percentage, and sunshine hours).
